# Newly designed solid coupling medium for reducing trapped air pockets during extracorporeal shock wave lithotripsy_ a phantom study

**DOI:** 10.1186/s12894-021-00847-y

**Published:** 2021-05-14

**Authors:** Chien-Sheng Wang, Ching-Chia Li, Wen-Jeng Wu, Wen-Chin Liou, Yusen Eason Lin, Wei-Chuan Chen

**Affiliations:** 1 Department of Urology, School of Medicine, College of Medicine, Kaohsiung Medical University Hospital, No.100, Shiquan 1st Rd., Sanmin Dist., Kaohsiung City 807, Taiwan; 2 Department of Surgery, St. Joseph Hospital, Kaohsiung City, Taiwan; 3 Graduate Institute of Human Resource and Knowledge Management, National Kaohsiung Normal University, Kaohsiung City, Taiwan; 4 CleanWave Medical Co., LTD, Kaohsiung City, Taiwan

**Keywords:** Coupling, Air pockets, Extracorporeal shock wave lithotripter

## Abstract

**Introduction:**

Air pockets between the lithotripter head and body surface are almost inevitably generated when applying a handful of gel onto the contact portion of the treatment head and that on the patient’s skin during coupling procedure. These air pockets can compromise the transmission of acoustic energy of shock wave and may significantly affect efficacy of stone disintegration. Comparing to conventional gel, this study aims to investigate efficacy of stone disintegration by using a proprietary isolation-coupling pad (“icPad”) as the coupling medium to reduce trapped air pockets during ESWL procedure.

**Method:**

In this phantom study, Dornier lithotripter (Delta-2 RC, Dornier MedTech Europe GmbH Co., Germany) was used with a proprietary gel pads (icPad, Diameter = 150 mm, Thickness = 4 mm and 8 mm). The lithotripter was equipped with inline camera to observe the trapped air pockets between the contact surface of the lithotripter head. A testing and measuring device were used to observe experimental stone disintegration using icPad and semi-liquid gel. The conventional semi-liquid gel was used as control for result comparison.

**Results:**

The stone disintegration rate of icPad 4 mm and 8 mm after 200 shocks of energy at level 2 were significantly higher than that of the semi-liquid gel (disintegration rate 92.3%, 85.0% vs. 45.5%, respectively, p < 0.001). The number of shocks for complete stone disintegration by icPad of 4 mm and 8 mm at the same energy level 2 were significantly lower than that of the semi-liquid gel (the number of shocks 242.0 ± 13.8, 248.7 ± 6.3 vs. 351.0 ± 54.6, respectively, p = 0.011). Furthermore, quantitative comparison of observed air pockets under Optical Coupling Control (OCC) system showed that the area of air pockets in semi-liquid group was significantly larger than that of the group using icPad (8 mm) and that of the group using icPad (8 mm) after sliding (332.7 ± 91.2 vs. 50.3 ± 31.9, 120.3 ± 21.5, respectively, p < 0.05).

**Conclusion:**

The advantages of icPad includes: (1) reduced the numbers of shock wave and increased stone disintegration rate due to icPad’s superior efficacy; (2) significantly reduce trapped air pockets in ESWL coupling. Due to the study limitation, more data are needed to confirm our observations before human trials.

## Introduction

There has been a marked development of lithotripsy techniques in the late 1990s or the early 2000, including precise localization (in-line navigation systems) with automatic ultrasound robotic arm, monitoring and stone fixation, implementation of different focal sizes with new acoustic lenses, coupling control, slower pulse rates, and ramping strategies [1–4]. Modern lithotripters are dry-head devices in which the treatment head is shifted into contact with the patient. The typical protocol for coupling is to apply a handful of gel onto the contact portion of the treatment head, and to the contact area on the patient’s skin. However, quality of coupling is usually not concerned by operators during ESWL procedure [5]. Entrapped air pockets can get caught at the coupling interface and impair the transmission of shock waves [6, 7]. Neucks et al. has conducted an in vitro study and discovered that air pockets covering 1.5–19% of coupling area result in a reduction in shock wave (SW) amplitude of 20%, and even 2% air coverage could decrease stone breakage rate by 20–40% [8]. Good quality of coupling, with minimal or no air trapped between the two contact surfaces, is the key factor to prevent transmission of acoustic energy loss and to improved efficacy of stone disintegration for ESWL.

Several strategies have been proposed to minimize air pockets in the coupling area. Coupling technique by applying a larger amount of semi-liquid gel to the lithotripter head leads to better coupling [8]. Bergsdorf et al. invented a coupling membrane and demonstrated better efficacy over semi-liquid gel, but not being widely adopted [9]. Lithotripter equipped with optical coupling control (OCC) system was invented. Under visual surveillance, the physician can halt and re-apply gels to treatment head if the air pockets are observed [10]. However, the high cost might hinder its widespread adoption. Thus, we conducted an observational study on the use of a single-use, proprietary isolation-coupling pad ("icPad") (Taiwan patent, utility model patent, M480354) as coupling medium to reduce trapped air pockets and increase efficacy of stone disintegration during ESWL procedure.

## Materials and methods

### Test medium

**Fig. 1:**
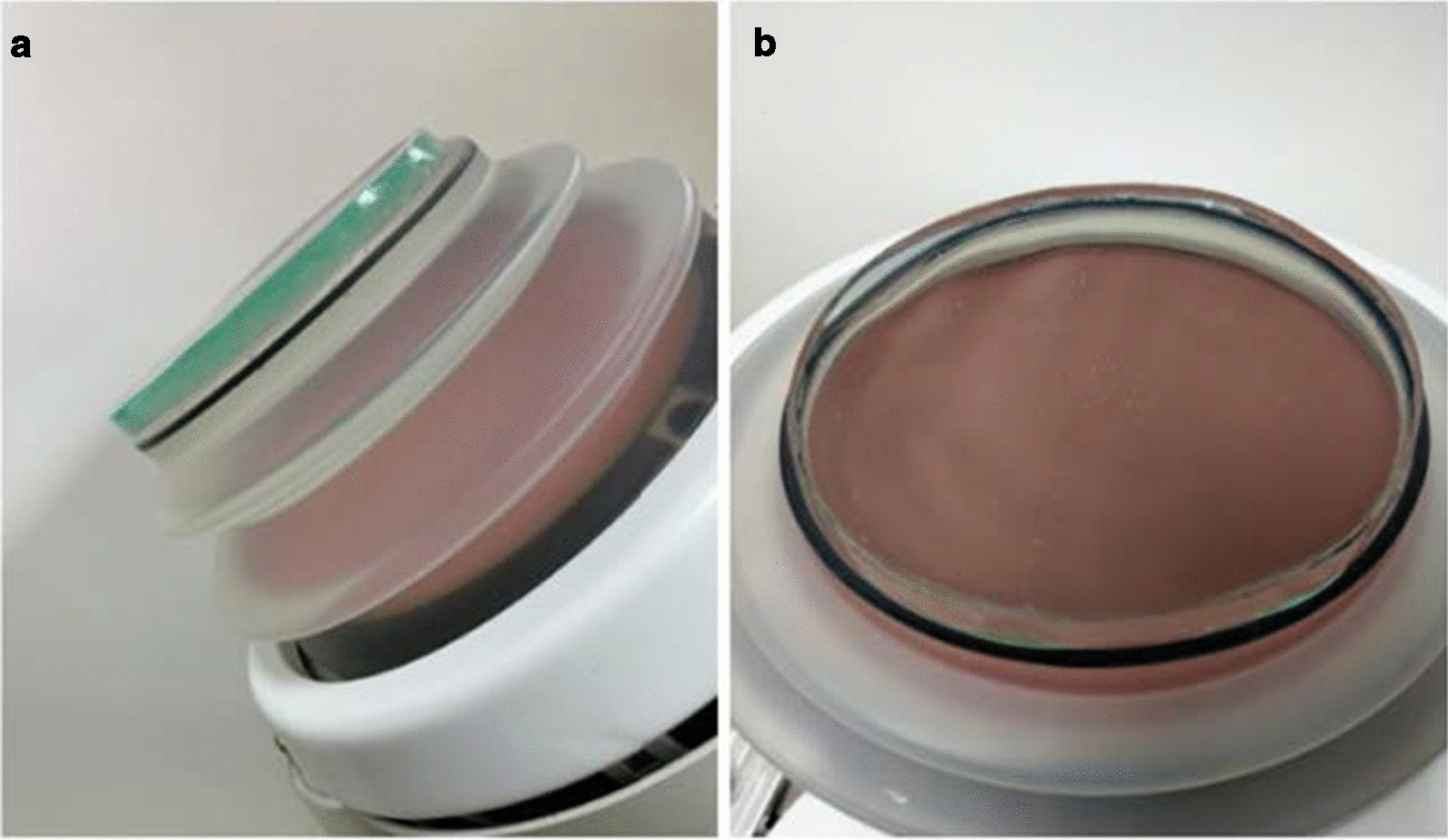
A proprietary icPad (light green color) sticking on the treatment head. **a** side view and **b** bottom view

**Fig. 2:**
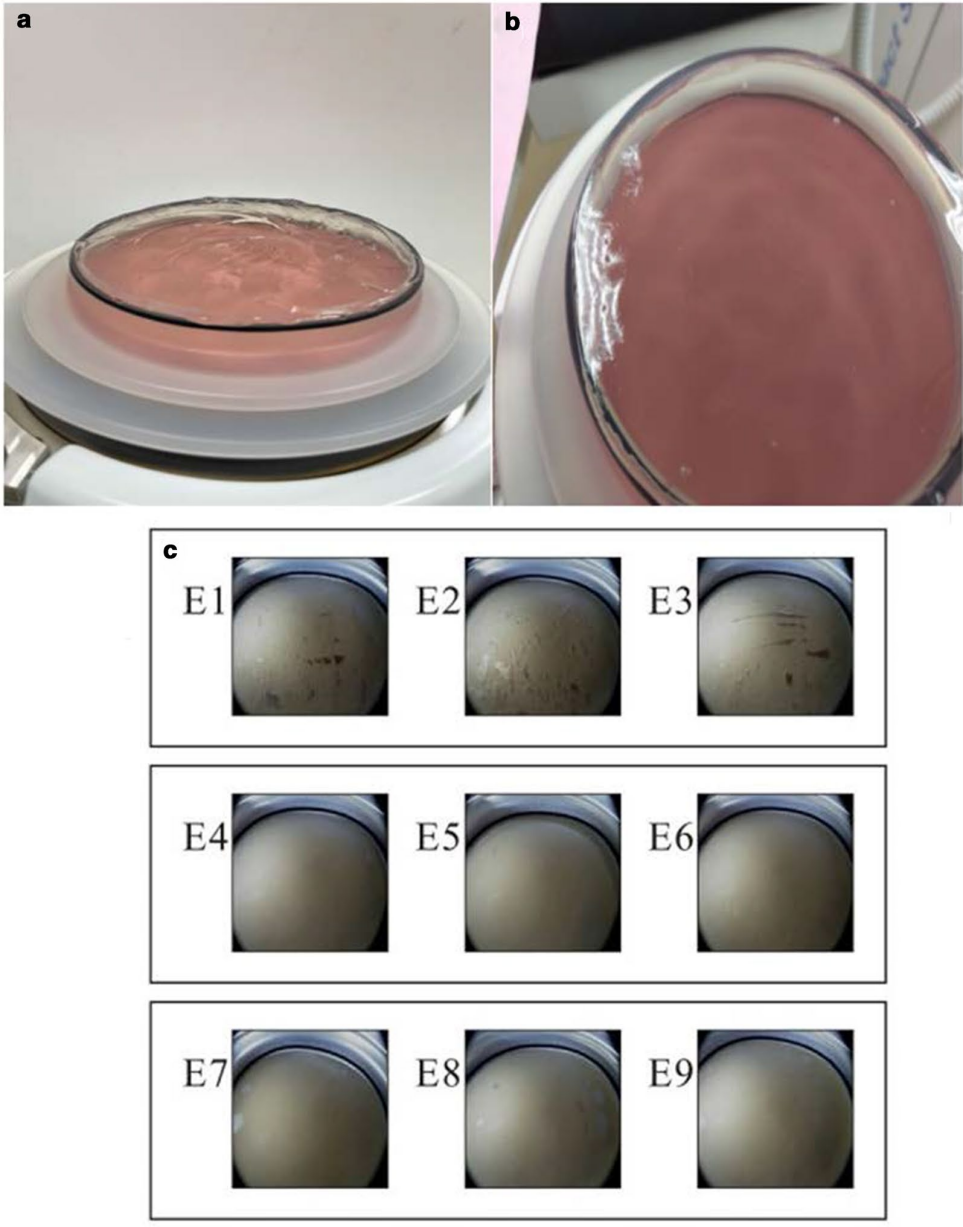
**a** Uneven surface and **b** smooth surface using semi-liquid gel and icPad on treatment head. **c** Comparisons of trapped air pockets between icPad and semi-liquid gel. Apparent air pockets were seen on experiments (E) using semi-liquid gel (E1–E3). No obvious air pockets were observed in the experiments using icPad (E4–E6) and remained almost air-free after sliding the icPad (E7–E9)

The medium tested are proprietary gel pads (icPad) (Diameter = 150 mm, Thickness = 4 mm and 8 mm) consisting of chemical-gel, mainly polyacrylamide, (Fig. 1) and standard semi-liquid gel (Sonogel®), which is widely used in clinical practice, as control. We used a clinical lithotripter (Delta-2 RC, Dornier MedTech Europe GmbH Co., Germany) equipped with an inline video camera with OCC (Optical Coupling Control) system for coupling monitoring. In the study group, we applied the icPad on the treatment head similar to the method we apply thin-film screen protector to a smart phone. The other side of icPad then was sprayed with specified liquid lubricant and attached to the water tank of the testing device in which the model stone was immersed. In the control group, we applied abundant amount of semi-liquid gel to the lithotripter head as Neucks et al. described to decrease the air pockets trapped shown on Fig. 2a, b [8]. If air pockets still existed under OCC system in both groups, we would reapply up to 3 times in our study protocol. Then, experiments were triplicated for each group. The procedure of sliding test was also performed to observe whether air pockets would be present after sliding the icPad. This procedure aimed to simulate patient’s involuntary body movement during localization of targeted stone and ESWL procedure.

### Experimental procedure

**Fig. 3:**
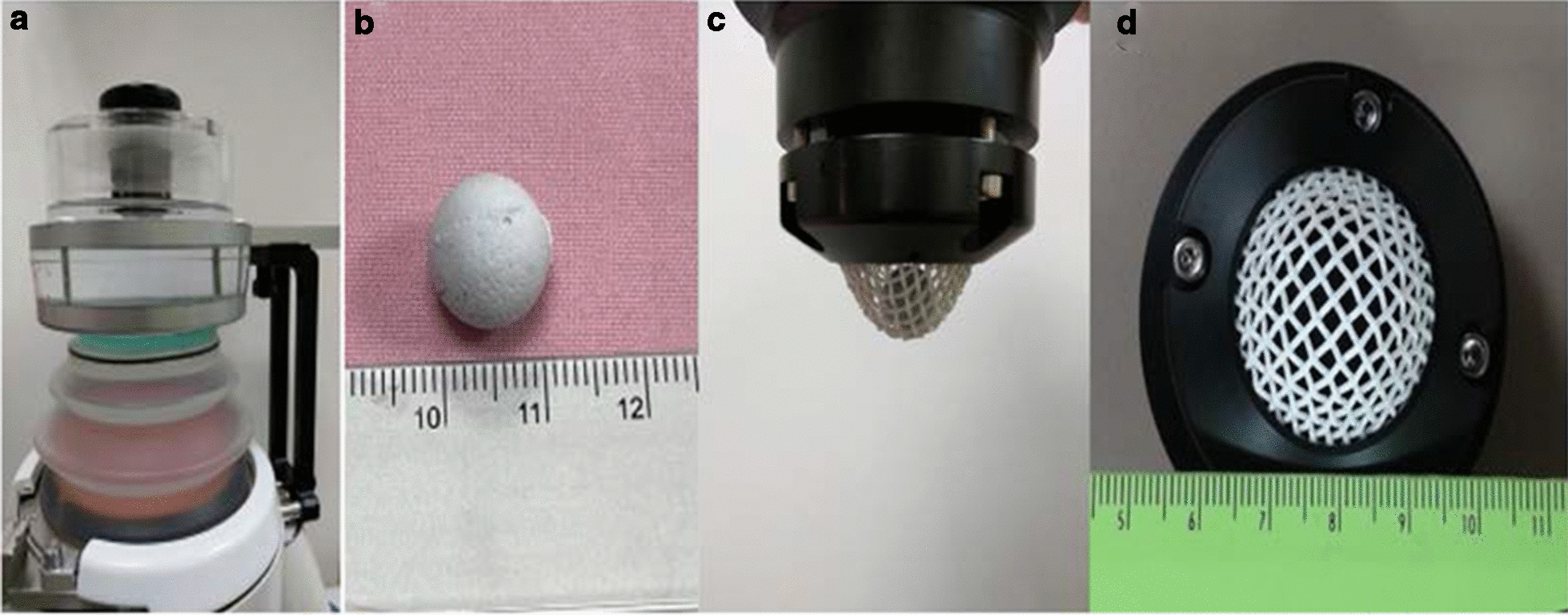
**a** A testing and measuring device containing **b** a model stone placed in the **c** basket with the **d** sieve size of 2 mm. The icPad (green part) placed between treatment head and water tank in which a model stone immersed shown on (**a**)

**Fig. 4:**
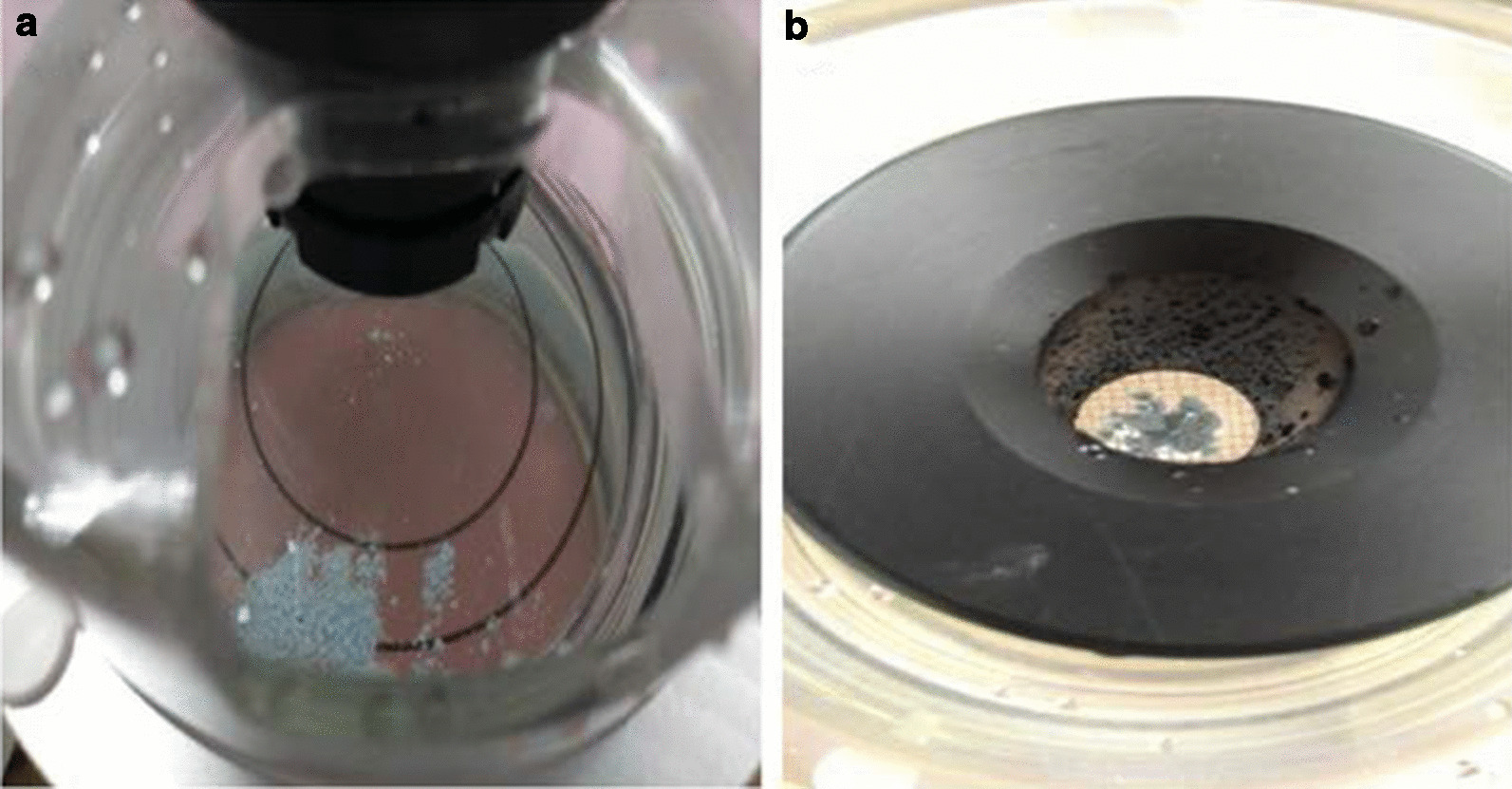
**a** Disintegrated stone fragments passed through the sieve after shock wave. **b** The fragments remained on the sieve

A testing and measuring device (Dornier MedTech Europe GmbH Co., Germany) and standardized model stones (K0742691, Dornier MedTech Europe GmbH Co., Germany) were used to measure the performance of stone disintegration of lithotripters (Fig. 3). Each model stone was weighted before disintegration and underwent 200 shocks at energy level 2(corresponding energy = 20 mJ, acoustic pressure range 50–90 MPa) at rate of pulse of shock of 80 per minutes. Two parameters, including disintegrated stone rate and number of shocks needed for complete stone disintegration, were measured for the efficacy of stone disintegration. First, fragments passed through the sieve and those which remained on the sieve (sieve size = 2 mm) were collected as residual portion after shock wave (Fig. 4). The residual fragments were later weighed after desiccation. Stone disintegration rate then was defined as the following formula:

$${\text{Stone disintegration rate}}=\frac{\text{Weight of model stone}-\text{Weight of residual stone}}{\text{Weight of model stone}}$$

Second, the number of shocks needed for complete stone disintegration was recorded when no visible residual stone fragments on the sieve during the test.

Quantitative comparison of observed air pockets between the contract surface and the lithotripter head was performed using icPad (8 mm) and semi-liquid gel. We calculated the irregular area covered by air pockets using imaging recognition (@5 × 5 pixel grid, Photoshop CS6) (Additional file 1: Figure S1).

### Statistical analysis

One-way ANOVA with post-hoc pairwise two-group comparisons were used for numerical variables. Statistical significance was set at *p* < 0.05. IBM SPSS 26.0 (IBM Corp., Armonk, NY) was used for all statistical analyses.

## Results

### Stone disintegration

The post hoc comparison showed that icPad 4 mm and 8 mm have significantly higher stone disintegration rate (icPad 4 mm 93.5%, icPad 8 mm 85.0%) than semi-liquid gel group (45.5%) (*p* < 0.001). The number of shocks needed for complete stone disintegration also revealed similar results: icPad 4 mm and 8 mm have significantly lower number needed for complete stone disintegration (icPad 4 mm 242.0, icPad 8 mm 248.7) than semi-liquid group (351.0) (*p* < 0.05), more than 20% faster than semi-liquid gel (Table 1).

**Table 1:** The results of disintegration rate after 200 shocks and number of shocks for complete stone disintegration for different test groups using icPad and semi-liquid gel

	Group	Mean ± SD	F value	Post-hoc Scheffé results
Stone disintegration rate (%) after 200 shocks	icPad 4 mm	92.3 ± 2.1	79.166***	Semi-liquid gel < icPad 4 mm and 8 mm
	icPad 8 mm	85.0 ± 7.5		
	Semi-liquid gel	45.5 ± 3.6		
Number of shocks for complete disintegration	icPad 4 mm	242.0 ± 13.8	10.446*	Semi-liquid gel > icPad 4 mm and 8 mm
	icPad 8 mm	248.7 ± 6.3		
	Semi-liquid gel	351.0 ± 54.6		

^*^p < 0.05; **p < 0.01; ***p < 0.001

### Air pockets observation

The amount of air pockets observed from OCC in icPad and semi-liquid gel were shown on Fig. 2c. Despite the attempt to reduce trapped air pockets under OCC system, there were still noticeable amount of air pockets (in dark-gray or bright color) observed from the camera in the group using semi-liguid gel (E1–E3). Almost air-free on the central area and few marginal air pockets were observed using icPad (E4–E6). The procedure of icPad sliding was performed on the testing device to simulate the patient’s body movement for localization of target stone during ESWL and the trapped air pockets remain the same (E7-E9).

The area of air pockets in semi-liquid (Fig. 2c E1–E3) is significantly larger that of the group using icPad (8 mm) (Fig. 2c E4–E6) and that of the group after sliding (Fig. 2c E7–E9)( 332.7 ± 91.2, 50.3 ± 31.9, 120.3 ± 21.5, respectively, *p* < 0.05) (Table 2).

**Table 2:** The results of quantitative comparison of observed air pockets between the icPad (8 mm) and semi-liquid gel

	Group	Mean ± SD	F Value	Post-hoc Scheffé results
Area of air pockets (pixel grid)	icPad 8 mm	50.3 ± 31.9 (0.38%)	16.051**	Semi-liquid gel > icPad 8 mm and 8 mm after sliding
	icPad 8 mm after sliding	120.3 ± 21.5 (0.92%)		
	Semi-liquid gel	332.7 ± 91.2 (2.55%)		

*p < 0.05; **p < 0.01; ***p < 0.001

## Discussion

Since the first procedure performed in 1980, ESWL has revolutionized the management of urolithiasis [11]. Ever since, several techniques of ESWL including shock wave generation, localization system, larger focal zone, ramping strategy, lower pulse rate, stone localization, and adequate coupling in lithotripter design have evolved to achieve better stone disintegration over the past four decades [12–14]. In this study, we proposed a new strategy to reduce the trapped air pockets during ESWL coupling by using a new acoustic, proprietary isolation-coupling pad (icPad). icPad exhibits higher efficacy and faster stone disintegration (Table 1). For example, under the same condition of 200 shocks, icPad group showed significant better efficacy than the semi-liquid gel (92.3% and 85.0% vs. 45.5%). Under the inline camera attached to the lithotripter, the trapped air pockets observed is only 0.38% in icPad groups over the treatment head, which is significantly lower than that of semi-liquid gel group (2.55%). Even sliding occurred, the air pockets only increased as little as 0.54%. Thus, icPad demonstrated better stone disintegration efficacy and lower air pockets in comparison with semi-liquid gel. In addition, our data support Neucks et al. and Jain et al. that the air pocket coverage over 1.5% could compromise the ESWL efficacy [6, 8]. In our study, icPad can maintain air pocket coverage rate below 1% and exhibits better ESWL efficacy.

When using icPad, three advantages are worth considering: coupling, adhesion and isolation. First, the transmission coefficient for an acoustic wave moving from water to air is only 0.1%, which means 99.9% of the wave will be reflected [15]. In our study, OCC system showed that we can achieve minimal air pockets (< 0.5%) at the coupling area when using icPad. Even after sliding, the air pockets only increased as little as 0.54%, comparing to the air pockets as much as 2.55% when using semiliquid gel. This may enhance the transmission of shockwave and improve ESWL efficacy. Second, icPad is a solid polyacrylamide gel which can firmly attach to the receiving object to ensure minimal air pockets in the coupling area. Third, from the practical infection control point of view, icPad provides a good isolation between lithotripter treatment head and the receiving object. However, this feature requires further experimental data under case-control clinical study design to support our hypothesis.

The limitation of this study is that we only demonstrate the correlation between ESWL efficacy and the presence of air pockets. In addition, we are unable to differentiate whether better ESWL efficacy is due to low air pockets or the nature of the solid coupling medium. Therefore, more data are needed to confirm our observations before human trials.

To our knowledge, this is the first evidence-based observational study showing that a solid gel pad (icPad) can minimize the trapped air pockets during ESWL, utilizing an inline camera for quantitative evaluation. The experimental results show that icPad has advantages in better efficacy in stone disintegration and lower air pockets.

## Supplementary Information

**Additional file 1: Fig. S1**. Area of irregular shape-air pockets was calculated by imaging recognition using Photoshop CS6 at 5x5 pixel grid (e.g. Fig. 2c E3).

## Data Availability

All data generated or analysed during this study are included in this published article and its supplementary information files.
