# Supplementary figures and images for: Newly designed solid coupling medium for reducing trapped air pockets during extracorporeal shock wave lithotripsy_ a phantom study
Source: BMC Urol. 2021 May 14;21:79. doi: 10.1186/s12894-021-00847-y (PMC8120698; doi:10.1186/s12894-021-00847-y)

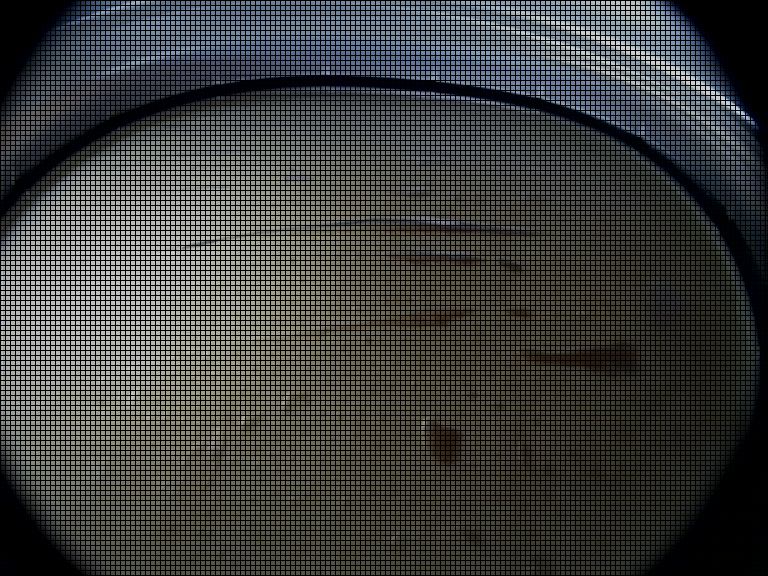

Supplement: Supplementary file 1 — Additional file 1: Fig. S1. Area of irregular shape-air pockets was calculated by imaging recognition using Photoshop CS6 at 5x5 pixel grid (e.g. Fig. 2c E3). [file 12894_2021_847_MOESM1_ESM.tif]
